# Dental Pulp Response to Silver-Containing Solutions: A Scoping Review

**DOI:** 10.3390/dj11050114

**Published:** 2023-04-26

**Authors:** Ahmed Zaeneldin, Chun-Hung Chu, Ollie Yiru Yu

**Affiliations:** Faculty of Dentistry, The University of Hong Kong, Hong Kong SAR, Chinachchu@hku.hk (C.-H.C.)

**Keywords:** silver fluoride, silver nitrate, silver diamine nitrate, silver diamine fluoride, nano-silver fluoride, dental pulp

## Abstract

Dentists used silver-containing solutions for deep cavity disinfection before restoration. This review aims to identify the silver-containing solutions reported in the literature for deep cavity disinfection and summarize their effects on dental pulp. An extensive search was performed using the search words “(silver) AND (dental pulp OR pulp)” in ProQuest, PubMed, SCOPUS, and Web of Science to identify English publications on silver-containing solutions for cavity conditioning. The pulpal response to the included silver-containing solutions was summarized. The initial search identified 4112 publications and 14 publications met the inclusion criteria. Silver fluoride, silver nitrate, silver diamine nitrate, silver diamine fluoride, and nano-silver fluoride were used in deep cavities for antimicrobial purposes. Indirect silver fluoride application induced pulp inflammation and reparative dentine in most cases, and pulp necrosis in some cases. Direct silver nitrate application caused blood clots and a wide inflammatory band in the pulp, whilst indirect silver nitrate application caused hypoplasia in shallow cavities and partial pulp necrosis in deep cavities. Direct silver diamine fluoride application induced pulp necrosis, while indirect silver diamine fluoride application induced a mild inflammatory response and reparative dentine formation. No evidence of the dental pulpal response to silver diamine nitrate or nano-silver fluoride was available in the literature.

## 1. Introduction

Dental caries remains one of the most prevalent diseases worldwide [1]. As the carious lesions progress, dental hard tissues are destroyed [2]. In advanced stages, the dentine–pulp complex can be involved [3]. Restorative treatment is indicated for carious teeth with moderate to advanced lesions.

Restorative treatment of carious teeth requires removing the carious tissues and restoring them with dental filling materials [4]. Because caries removal in deep lesions during restorative treatment implies a higher risk of pulp exposure or pulp vitality loss, the concept of selective or partial caries removal is now commonly adopted [4,5,6]. Selective caries removal made the “extension for prevention” concept, by G.V. Black, and the conventional non-selective caries removal obsolete in preserving the natural tooth structure and pulp vitality [7]. In the selective caries removal technique, the inner part of the caries lesion is preserved while the superficial part of the caries lesion is removed and restored with restoration [8]. This concept is based on the histological finding that carious dentine can be divided into two structurally distinct zones [9]. The superficial layer is known as infected dentine, which is irreversibly demineralised dentine consisting of cariogenic microorganisms, metabolic by-products, and degraded collagen networks due to proteolytic degradation [10]. The inner layer is known as the affected dentine, which is reversibly demineralised dentine consisting of acid-demineralized minerals and a repairable collagen network [11].

However, it is almost impossible to confirm that all the infected dentine-containing cariogenic microorganisms are removed in the clinical situation. The infected dentine and the affected dentine are difficult to differentiate clinically. Currently, the most common strategy to differentiate the infected and affected dentine is to use tactile sensations to determine the hardness during caries excavation, which is liable to subjective judgement [12]. In some cases, a dense portion of the microorganisms might be left in the cavity after excavation, resulting in recurrent caries.

The elimination of the microorganisms in the cavity is the key to the success of selective caries removal and the subsequent restoration. The elimination of the microorganisms can be achieved by an ideal sealing of the cavity with the restorative materials. The sealed microorganisms would be isolated from the oral environment without nutrients and a biofilm would not form [13]. However, ideal sealing can be difficult to achieve or maintain with current restorative materials. Using antimicrobial agents for cavity conditioning or lining can kill the cariogenic bacteria and inhibit the further development of cariogenic biofilm in the cavity, reducing the risk of secondary caries and potentially improving the longevity of the restoration.

Silver is a broad-spectrum antimicrobial agent that can be used for caries management [14]. Resistance to the antimicrobial action of silver has seldom been found in bacteria [15,16]. The effect of silver-containing solutions in caries control has been confirmed in previous studies [17,18,19]. The mechanism of silver’s antimicrobial action is closely related to its interaction with the thiol (sulfhydryl) groups in the cariogenic bacteria [20]. Silver interacts with the enzymes and proteins in the cell by combining with the thiol groups, which are key functional groups in the enzymes [21]. The silver ions can release the potassium ions from the bacterial cytoplasmic membrane, which is a site associated with numerous enzymes [20]. Moreover, silver can cause the marked inhibition of cell growth and damage the cell envelope as silver granules are deposited in the vacuoles and cell walls [22]. Structural abnormalities and an increase in bacterial cell size have also been found. Finally, it can interact with the bases in the deoxyribonucleic acid (DNA) [23].

Because of their long-lasting antimicrobial effect, silver-containing solutions have been used for cavity disinfection after caries removal [24]. However, the effect of silver-containing solutions on dental pulp beneath deep cavities has never been elucidated. Therefore, this scoping review aims to identify and provide a comprehensive overview of the available silver-containing solutions for deep cavity disinfection and summarize their effects on dental pulp.

## 2. Materials and Methods

**Figure 1:**
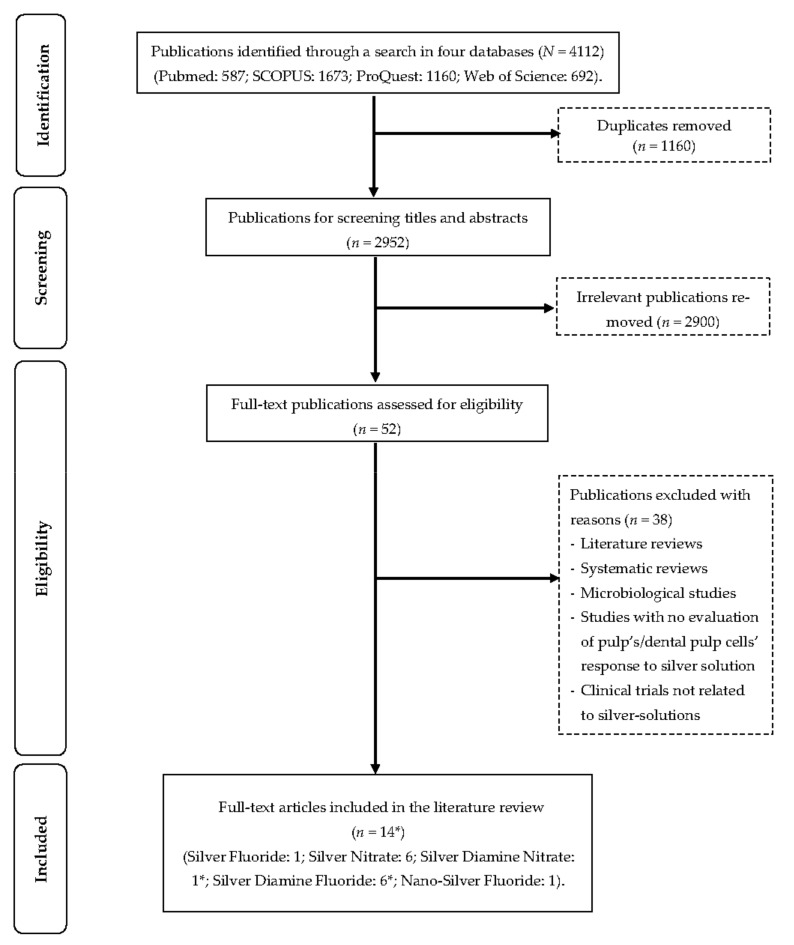
Flow diagram of literature search. * *One study has both silver diamine nitrate and silver diamine fluoride*.

This review was prepared according to the PRISMA checklist for scoping reviews [25]. An extensive search was performed using the search terms “(silver) AND (dental pulp OR pulp)” in four databases (ProQuest, PubMed, SCOPUS, and Web of Science). The included studies were limited to articles published in English on or before 1 November 2022. Duplicate articles; clinical, in vivo, or in vitro studies not related to silver-containing materials; abstracts; conference papers; literature reviews; and systematic reviews were excluded. For a study to be included, it had to evaluate the pulpal response (clinical, in vivo, and ex vivo) or the cell’s pulpal response (in vitro) to silver-containing materials used as cavity conditioners. Two authors participated in the study selection process (A.Z. & O.Y.Y.), and in case of a disagreement, they referred to a third author (C.-H.C.). Important information was collected on separate spreadsheets by the investigators. All the reference lists of the included studies were checked for potential studies. The study selection process is presented in the flow diagram below (Figure 1). A summary of all the included studies can be found in Table 1.

## 3. Silver-Containing Solutions for Deep Caries Management

The main properties of various silver-containing solutions are summarized in Table 2.

### 3.1. Silver Fluoride

The 40% silver fluoride solution was used for deep caries management. The 40% silver fluoride solution contained 34% silver and 6% fluoride. According to this percentage, the fluoride content was supposed to be 59,900 ppm [40]. The chemical analysis of the components of two silver fluoride products found that the fluoride content was higher than expected, ranging from 78,000 to 120,000 ppm [40,41]. As a result of these findings, Gotjamanos & Afonso [41] recommended that the use of silver fluoride in paediatric dentistry be discontinued because these unacceptably high fluoride levels pose a potential risk of toxicity to children and, if it enters the bloodstream, it might result in fluorosis, especially in the case of an undetected pulp exposure [42]. Silver fluoride has antibacterial and remineralising properties. The silver ions present in the silver fluoride act in two different ways: (1) their bactericidal/bacteriostatic effect on the microorganisms present in the carious lesion; (2) mechanical sealing of the carious and sound dentinal tubules [26]. Silver fluoride is used as a cavity conditioner in conjunction with glass ionomer cement as an atraumatic restorative method. After partial caries excavation, silver fluoride is applied, and then the cavity is sealed with a glass ionomer cement restoration. Silver fluoride showed a 100% success rate in 400,000 cases treated at the Dentistry School of Western Australia University [26]. The treatment success was based solely on the absence of symptoms.

### 3.2. Silver Nitrate

The first use of silver nitrate was reported around 1846 [41]. The results, reported by Stebbins in 1891, shed light on the clinical significance of silver nitrate [43]. It was mainly used to arrest dental caries and sterilize cavities because of its escharotic, dehydrating, and sclerosing properties [16]. Silver nitrate is acidic in nature, and it causes protein coagulation [44]. In 1917, Howe introduced ammoniacal silver nitrate by adding ammonium hydroxide to silver nitrate [45]. The addition of the ammonium hydroxide converted the acidic silver nitrate into a solution with an alkaline nature, limiting its irritating action [41]. The new compound, which contained 25% silver nitrate, was later known as Howe’s solution and was widely used by practitioners.

The nature of dental caries as a noncommunicable disease rather than an infectious disease made the idea of the sole application of silver nitrate debatable. The results of various studies regarding the efficacy of silver nitrate in the prevention of dental caries have not revealed any significant reduction in caries incidence compared to no treatment [46,47,48]. Other studies examined the efficacy of silver nitrate in arresting carious lesions and reported that it can arrest caries on both permeant and primary dentition [48,49]. The use of silver nitrate in preventing/arresting dental caries decreased significantly after fluoride was introduced [16]. In 2015, silver nitrate was used in conjugation with sodium fluoride, and the reported results exhibited effectiveness in arresting dental caries [50].

### 3.3. Silver Diamine Nitrate

Silver diamine nitrate was developed from silver nitrate. The 48% silver diamine nitrate solution was proposed to act as a less expensive alternative to silver diamine fluoride in regions with lower and middle-income economies as it lacks the fluoride component. There is a delay in the mineral induction time of silver nitrate, which affects its remineralization potential [51]. The addition of the diamine group to silver nitrate stabilizes the silver ions, and it is expected to enhance its mineralization potential [33]. When the silver diamine nitrate is applied to a dentine surface, it reacts with the ions present in the environment and produces silver phosphate and silver oxide. The silver compounds may readily react with the chloride in the environment, resulting in the formation of less soluble silver chloride [52,53]. In the presence of silver salts, demineralized dentine becomes harder and dentinal tubules become blocked, preventing any further progress of the acidic by-products responsible for demineralization [52]. The efficacy of silver diamine nitrate in caries control has not yet been reported.

### 3.4. Silver Diamine Fluoride

Introduced by Dr Nishino and Dr Yamaga in the 1950s, silver diamine fluoride (SDF) has been used for arresting caries, preventing secondary caries, and decreasing hypersensitivity [54]. Commercial SDF products are available in various concentrations (3.8%, 10%, 12%, 30%, and 38%). Solutions in all of these concentrations are suitable for use as anticaries solutions, with the exception of the 3.8% solution, which is intended for root canal therapy. Silver diamine fluoride is an alkaline solution with a high concentration of fluoride and silver ions [1]. Fluoride changes the hydroxyapatite into fluorapatite, which is more resistant to acid dissolution, so it can remineralise the caries-affected dental hard tissues [15]. In addition, it has an antimicrobial effect on the plaque biofilm [55]. Silver, the other main component of silver diamine fluoride, is well known for its strong antimicrobial effect which helps in sterilizing the infected tissues, and it blocks the dentinal tubules to seal them from microorganisms and their by-products [56]. Combing both silver and fluoride in one solution so they can perform their actions synergistically resulted in the superior results achieved by the silver diamine fluoride. Despite its effectiveness, silver diamine fluoride usage for arresting caries is still an off-label usage in many countries, where it is used only as a desensitizing agent. Silver diamine fluoride requires only topical application to achieve its effect. This ease of use has led to its widespread use among practitioners recently, especially during the COVID-19 pandemic [57]. The World Health Organization’s Center for Quality Improvement and Evidence-Based Dentistry introduced safer aerosol-free emergent dentistry as a protocol for caries management [58]. Silver diamine fluoride was the first choice in this protocol for the treatment of caries and toothache related to caries, as its use can prevent cross-infection. The effectiveness of silver diamine fluoride in caries control has been proved in many previous studies [59,60,61].

### 3.5. Nano Silver Fluoride

Silver nanoparticles have been introduced in dentistry for multiple applications, including caries management [62]. The antibacterial effect of silver nanoparticles relies on their large contact area with the microorganisms due to their nanometric particle size [63]. Silver ions can be continuously released by the silver nanoparticles [64]. This disrupts the permeability and respiration of the bacteria by binding to the sulphur proteins in the cytoplasmic membrane and cell wall [65,66,67]. A further instance of this is the detection of reactive oxygen species inside bacterial cells. Reactive oxygen species elevate the oxidative stresses and damage the deoxyribonucleic acid (DNA) [67,68]. In addition, the interaction of the DNA’s sulphur and phosphorus, with silver ions, can create issues with DNA replication and cell reproduction, and can even cause microorganism death [69]. The denaturation of the cytoplasmic proteins is another effect of silver ions [70]. Moreover, silver nanoparticles can kill bacteria themselves by accumulating in the pits that form the cell wall after attaching to the cell surface [71]. Furthermore, silver nanoparticles can disrupt bacterial signal transduction, resulting in a decrease in proliferation and cell apoptosis [72]. Fluoride solutions, combined with silver nanoparticles, proved the capability to remineralise carious lesions [73]. Therefore, silver nanoparticles, in the form of nano-silver fluoride, have been developed for caries management [74]. The effectiveness of nano-silver fluoride as a cariostatic agent was investigated by dos Santos et al. [75]. They found that active caries decreased by 50% at 12 months after a single application of nano-silver fluoride [75]. Nagireddy et al. conducted another study in which nano-silver fluoride was applied to dentinal caries, and 65.21% of the carious lesions were arrested after a 1-year follow-up [76].

**Table 2:** Properties of silver-containing solutions for deep caries management.

Solution [Ref.]	Wt.% (Concentration According to Manufacturer(s))	Anticaries Properties
Silver fluoride [40,42]	40% (Ag: 340,000 ppm; F: 60,000 ppm)	- Inhibits growth of cariogenic bacteria - Prevents demineralization - Promotes remineralization
Silver nitrate [77]	25% (Ag: 151,130 ppm)	- Inhibits growth of cariogenic bacteria
Silver diamine nitrate [33]	48% (Ag: 319,914 ppm *)	- Inhibits growth of cariogenic bacteria
Silver diamine fluoride [78]	12% (Ag: 80,170 ppm; F: 14,150 ppm)	- Inhibits growth of cariogenic bacteria - Prevents demineralization - Promotes remineralization
	30% (Ag: 200,400 ppm; F: 35,400 ppm)	
	38% (Ag: 253,900 ppm; F: 44,800 ppm)	
Nano-silver fluoride [39]	1.05% * (Ag: 399.33 ppm; F: 10,147 ppm)	- Inhibits growth of cariogenic bacteria - Prevents demineralization - Promotes remineralization

* By calculation.

## 4. Dental Pulpal Response to Silver-Containing Solutions

Dental pulpal response to different silver-containing solutions is summarized in Table 3.

### 4.1. Silver Fluoride

Only one study reported the dental pulpal response to silver fluoride. In the study, by Gotjamanos [26], the 40% silver fluoride was applied as a cavity liner on the surface of residual caries in the deep proximal cavities. Fifty-five teeth were included in this study, and only five exhibited an unfavourable pulpal response. These five teeth displayed signs of acute and chronic inflammation, internal resorption, and necrosis. In contrast, the pulps of the other 40 teeth in the same study exhibited a favourable pulpal response with the presence of reparative dentine, a continuous odontoblast layer, no signs of tissue disarrangement, and no necrotic foci. The dental pulp response to various silver-containing solutions is summarized in Table 3.

### 4.2. Silver Nitrate

Six studies reported the dental pulpal response to silver nitrate [27,28,29,30,31,32]. In the study by Englander et al. [30], the indirect application of the silver nitrate on the dental pulp resulted in inflammatory changes, atrophy, and the destruction of the odontoblastic layer beneath the area of application. Many of the degenerating odontoblasts were found filled with silver. In addition, black silver particles were found inside the pulp, and haemorrhaging and oedema were observed in areas of dense silver accumulation. Despite these changes, the pulp tissue was normal in the deeper portions. In the same study, silver nitrate was applied directly to exposed areas of the dental pulp. The direct application of silver nitrate caused a large superficial haemorrhage that subsequently formed a blood clot. Large black globules of free silver were present on the surface of this blood clot, while the deeper portion of the clot was free of any globules. A wide inflammatory band was present beneath the clot, but the pulp beneath the area of inflammation was normal.

Perreault et al. [29] applied the silver nitrate to cavities at different depths. Hypoplasia occurred in the pulp under the shallow cavities, whereas complete pulp necrosis was found in the moderate and deep cavities. Concerns about the adverse side effects on the pulp were raised due to its high penetration ability [28,30]. Some researchers consider the presence of a sound dentine barrier between the carious lesion and the pulp crucial to limiting the silver nitrate’s cytotoxic effects [28,41,79].

### 4.3. Silver Diamine Nitrate

Only one study reported the dental pulpal response to silver diamine nitrate. In the in vitro study by Srisomboon et al. [33], the silver diamine nitrate was applied directly to human dental pulp stem cells, and the results showed only a 13% reduction in vitality. According to ISO 10993–5:2009, a reduction in cell viability by more than 30% is considered a cytotoxic effect. Silver diamine nitrate is therefore considered a non-cytotoxic material, though it contains a high concentration of silver [80]. Studies reporting the pulp response to silver diamine nitrate are scarce. No clinical studies on pulp response to silver diamine nitrate are available.

### 4.4. Silver Diamine Fluoride

Five studies reported the dental pulpal response to silver diamine fluoride. The in vitro study by Hu et al. [38] evaluated the cytotoxicity of silver diamine fluoride on dental pulp stem cells with direct and indirect applications. In the case of direct application, SDF was found to be toxic even at a very low concentration of 0.001% (silver: 253.87 ppm, fluoride: 44.8 ppm). Additionally, in the case of indirect application, there was 100% cell death with dentine discs 0.5 and 1 mm thick. With a dentine disc 1.5 mm thick, only 30% of the cells were vital. On the contrary, another in vitro study reported that 92% of dental pulp stem cells remained vital after a direct contact test with the silver diamine fluoride [33].

The histological findings of the ex vivo study by Korwar et al. [35] showed that there were no inflammatory signs and the tertiary dentine was formed after using silver diamine fluoride as a cavity conditioner. Another ex vivo study used silver diamine fluoride for indirect pulp capping in both an animal and a human model [36]. The results in both models showed mild inflammatory signs and the tertiary dentine was formed. Moreover, a case study by Bimstein and Damm [37] confirmed the same findings. The results of these studies are all in agreement that there was no irreversible damage to the pulp tissue after the silver diamine fluoride was used as an indirect capping agent, and it even promoted tertiary dentine formation [35,36,37]. Hosoya et al. [34] applied silver diamine fluoride directly on the exposed pulp of dogs’ teeth. The pulp in that study exhibited suppurative inflammation, round cell infiltration, bleeding, hyperaemia, and partial necrosis.

The in vitro study by Luong et al. [81] evaluated ion penetration into the pulp chamber after the application of various silver diamine fluoride products. Their results showed that the amount of silver and fluoride that can reach the pulpal space is low, and it lies within the safety limit. The results of this study must be interpreted carefully, as it did not provide information such as the location of the SDF application within the tooth, the remaining dentine thickness, or the absence of carious lesions.

### 4.5. Nano Silver Fluoride

Only one study reported the biocompatibility of nano-silver fluoride in human erythrocytes; no study on dental pulp cells was found. Targino et al. [39] evaluated the nano-silver fluoride’s cytotoxicity based on its haemolytic activity in human erythrocytes in vitro. The nano-silver fluoride was not toxic at any tested concentration (100, 50, 25, 12.5 and 6.25 µg/mL) in this study. We did not find any studies that have evaluated the dental pulp cell’s response (e.g., odontoblasts, odontoblast-like cells, fibroblasts, and dental pulp stem cells) to nano-silver fluoride. Concerns still arise around the increased daily exposure to silver nanoparticles, as they are incorporated into various aspects of life. There are not enough in vitro biological studies to evaluate their effect on the pulpal cells on the molecular level.

## 5. Limitation

Only articles published in English or an English translation, which was provided by their authors, were included in this review. The silver-containing solutions have been commonly used in non-English-speaking countries, such as Argentina, Japan, and Brazil, for many years. It is important to note that most articles published in these countries concerning silver-containing solutions are in languages other than English and are not indexed in the English databases. Consequently, these articles were difficult to access, and the incorporation of silver-containing solutions into dental treatment protocols in other countries was delayed. As an example, it was not until 2014 that silver diamine fluoride was introduced to the United States of America, despite having been used in Japan since 1969 [24]. Additionally, there was a lack of information regarding the biological effect of some of the included materials due to either their limited availability in the literature or their novelty. Additionally, the scope of scoping reviews is generally broad in scope, which limits the depth of analysis, and they do not include the risk of bias or any other assessments of the studies included. It is worth noting that scoping reviews are not intended to provide a definitive answer to a research question. It is imperative that more research be conducted on these materials due to the recent expansion of their use in different countries and their inclusion in treatment protocols in recent years.

## 6. Summary

Silver fluoride, silver nitrate, silver diamine nitrate, silver diamine fluoride, and nano-silver fluoride are silver solutions that can be used in carious cavities for antimicrobial purposes. Only the indirect silver diamine fluoride application induced a favourable pulpal response manifested as a mild inflammatory response and reparative dentine formation. No evidence of the dental pulpal response to silver diamine nitrate and nano-silver fluoride were available in the literature. This review underscores the importance of continued research in this area and the potential for future studies to contribute to our understanding. It also provides a guide in the clinical decision-making process. Although there is a growing interest in this topic, the insufficient number of studies makes it difficult to draw a definitive conclusion. Despite this, promising trends in the research indicate that data availability may increase in the future. Future investigations are warranted to explore the dental pulpal response to these silver solutions to support their usage as a cavity conditioner in case of deep cavities.

## Figures and Tables

**Table 1:** Summary of all included studies.

Solution	Author, Date	Study Type/Evaluation Method	Main Findings
Silver fluoride	Gotjamanos, T., 1996 [26]	In-vivo study/Histopathology	Favourable pulpal response, including the presence of abundant reparative dentine and a wide odontoblast layer in most teeth.
Silver nitrate	Zander, H., 1943 [27]	In-vivo & ex-vivo/Histology	Slight haemorrhage opposite to the cavity and silver particles within the pulp.
	Zander, H., 1945 [28]	In-vivo & ex-vivo/Histology	Odontoblast injury, silver particles within the pulp, and dentine formation.
	Perreault, J., 1956 [29]	In-vivo/Histopathology	Hypoplasia in shallow cavities, aplasia, and pulpal necrosis in deeper cavities.
	Englander, H., 1956 [30]	Ex-vivo/Histopathology	Silver particles penetrated through dentine into the pulp.
	Seltzer, S., 1961 [31]	In-vivo/Histopathology	Morphologic changes of the odontoblasts after 1 month (Monkeys). No pulpal effect after 1 month (Dogs).
	Seltzer, S., 1961 [32]	In-vivo/Histopathology	Pulp healed in shallow cavities but not in deep cavities after 6 months (Monkeys). Pulp healed in shallow cavities but inflammation in deep cavities after 6 months (Dogs).
Silver diamine nitrate	Srisomboon, S., 2022 [33]	In-vitro/Cytotoxicity	Cellular vitality was 88%.
Silver diamine fluoride	Hosoya, Y., 1990 [34]	In-vivo/Histopathology	Inflammation, hyperaemia, necrosis after 3 days. Hyperaemia, partial necrosis after 7 days. A decrease in inflammation, but an increase in pulp necrosis after 30 days.
	Korwar, A., 2015 [35]	Ex-vivo/Histopathology	Tertiary dentine formation with no inflammation or necrosis After 6 weeks.
	Rossi, G., 2017 [36]	Ex-vivo & in-vivo/Histopathology	Ex-vivo: Limited penetration of silver diamine fluoride into the dentinal tubules, chronic inflammatory infiltrate in the pulp tissue, and tertiary dentine formation. In-vivo: Well-organized dental pulp with mild inflammatory infiltrate.
	Bimstein, E., 2018 [37]	Case report/Histopathology	Tertiary dentine formation, minimal pulpal inflammation, and silver particles within dentinal tubules after 6 months.
	Hu, S., 2022 [38]	In-vitro/Cytotoxicity	Direct contact: Cellular viability was less than 50% above 0.001% silver diamine fluoride. Indirect contact: No vital cells for dentine 1 mm thick and 30% cell viability for dentine of 1.5 mm thick.
	Srisomboon, S., 2022 [33]	In-vitro/Cytotoxicity	Cellular vitality was 92%.
Nano-silver fluoride	Targino, A., 2014 [39]	In-vitro/Cytotoxicity	Non-toxic to human erythrocytes at any concentration.

**Table 3:** Dental pulpal response to different silver-containing solutions.

Solution	Pulpal Response to Direct Contact	Pulpal Response to Indirect Contact
Silver fluoride	- Not reported	- Signs of acute and chronic inflammation - Internal resorption - Necrosis - Formation of reparative dentine - Continuous odontoblast layer - No signs of tissue disarrangement
Silver nitrate	- Large superficial haemorrhage - Large black silver globules on blood clot surface - A wide inflammatory band beneath blood clot - Normal pulp beneath the area of inflammation	- Hypoplasia in shallow cavities - Complete pulp necrosis in deep cavities - Odontoblasts were filled with silver - Silver particles in the pulp - Normal pulp tissue in the deeper portion
Silver diamine nitrate	- Not reported	- Not reported
Silver diamine fluoride	- Hyperaemia - Round cell infiltration - Suppurative inflammation - Partial necrosis - Bleeding in the pulp tissue	- Chronic inflammation of pulpal tissue - Tertiary dentine formation - Well-organized pulpal structure - No bacteria in the pulp - Silver particles in the pulp
Nano-silver fluoride	- Not reported	- Not reported

## Data Availability

Not applicable.
